# A novel de novo *CASZ1* heterozygous frameshift variant causes dilated cardiomyopathy and left ventricular noncompaction cardiomyopathy

**DOI:** 10.1002/mgg3.828

**Published:** 2019-07-03

**Authors:** Jun Guo, Zheng Li, Chanjuan Hao, Ruolan Guo, Xuyun Hu, Suyun Qian, Jiansheng Zeng, Hengmiao Gao, Wei Li

**Affiliations:** ^1^ Beijing Key Laboratory for Genetics of Birth Defects, Beijing Pediatric Research Institute, MOE Key Laboratory of Major Diseases in Children, Genetics and Birth Defects Control Center, Beijing Children's Hospital Capital Medical University, National Center for Children's Health Beijing China; ^2^ Henan Key Laboratory of Pediatric Inherited & Metabolic Diseases, Henan Children's Hospital Zhengzhou Hospital of Beijing Children's Hospital Zhengzhou China; ^3^ Pediatric Intensive Care Unit, Beijing Children's Hospital Capital Medical University, National Center for Children's Health Beijing China

**Keywords:** *CASZ1* variant, DCM, LVNC, whole‐exome sequencing

## Abstract

**Background:**

Dilated cardiomyopathy (DCM) is the most common cardiomyopathy with a common presentation of heart failure. It has been reported that *CASZ1* loss‐of‐function mutation contributes to familial DCM and congenital ventricular septal defect (VSD). To date, only two pathogenic variants in *CASZ1* have been previously reported worldwide.

**Methods:**

To identify the causative variant in an 11‐month‐old Chinese boy with DCM and left ventricular noncompaction cardiomyopathy (LVNC), trio‐whole‐exome sequencing was performed followed by mutational analysis and Sanger sequencing.

**Results:**

An unreported de novo heterozygous frameshift variant (c.2443_2459delGTGGGCACCCCCAGCCT, p.Val815Profs*14) in *CASZ1* was idenitified in the proband. The frameshift mutation in *CASZ1* not only led to DCM but also presented an LVNC phenotype.

**Conclusion:**

We have identified a novel *CASZ1* variant in a patient with combined DCM and LVNC for the first time, thus broadening the phenotypic spectrum of *CASZ1* variants. Furthermore, this study emphasized the usefulness of whole‐exome sequencing for genetic diagnosis of cardiomyopathy.

## 1 INTRODUCTION

Dilated cardiomyopathy (DCM) is the most common cardiomyopathy, characterized by dilatation and impaired contractility of the left or both ventricles, and its common presentation is heart failure (HF) (Bharucha et al., 2015; Richardson et al., 1996). Recently, extrapolation of epidemiological data for DCM and HF suggests a prevalence as high as 1:250 in the general population worldwide (Hershberger, Hedges, & Morales, 2013). Left ventricular noncompaction cardiomyopathy (LVNC) is an increasingly recognized type of cardiomyopathy, characterized by a 2‐layered myocardium, numerous prominent trabeculations, and deep intertrabecular recesses communicating with the left ventricular (LV) cavity (Jenni, Rojas, & Oechslin, 1999; Oechslin, Attenhofer Jost, Rojas, Kaufmann, & Jenni, 2000). The combined incidence of DCM and LVNC was 0.77 per 100,000 person years in Sweden (Fadl, Wahlander, Fall, Cao, & Sunnegardh, 2018).

Dilated cardiomyopathy frequently has a genetic etiology and approximately 40% patients with familial DCM have an identifiable genetic origin (Ganesh et al., 2013). DCM has been reported in association with mutations in >100 genes, which encode molecules that participate in sarcomere integrity, cytoskeletal and nuclear architecture, force generation, force transmission, electrolyte homeostasis, gene transcription and mitochondrial function (Burke, Cook, Seidman, & Seidman, 2016). There is evidence for genetic overlap among DCM and LVNC. To date, more than 40 genes have been implicated in LVNC (Finsterer, Stollberger, & Towbin, 2017).

*CASZ1* is a para‐zinc‐finger transcription factor that is required for vertebrate heart development (Christine & Conlon, 2008). To date, only two pathogenic variants of *CASZ1* have been reported. A *CASZ1* missense mutation p.L38P has been identified in a family with congenital ventricular septal defect (VSD) (Huang et al., 2016), and a *CASZ1* nonsense mutation p.K351X has been identified to be associated with DCM (Qiu et al., 2017). Here we present the clinical features of a patient with a de novo *CASZ1* frameshift variant, c.2443_2459delGTGGGCACCCCCAGCCT (p.Val815Profs*14). The patient manifested DCM, LVNC and sudden cardiac arrest (SCA).

## 2 MATERIALS AND METHODS

### 2.1 Ethical compliance

The study was approved by the Ethics Committee of Beijing Children's Hospital, Capital Medical University.

### 2.2 Patient information

After obtaining informed consent, clinical data, pedigree information, blood samples were collected from the proband and his parents. All subjects underwent detailed physical evaluations by an experienced pediatrician, including blood examination, echocardiographic examination, electrocardiography (ECG), radiographs of chest, and other necessary tests.

### 2.3 Whole‐exome sequencing

DNA was extracted from peripheral blood samples of the proband and his parents using a QIAamp DNA Blood Mini Kit (Qiagen, Hilden, Germany) in accordance with the manufacturer's protocol. Trio‐whole‐exome sequencing was performed using the Agilent SureSelect Human All Exome V6 kit (Agilent Technologies Inc. Mississauga, ON, Canada) on an Illumina Hiseq X Analyzer (Illumina, San Diego, CA) with 150‐bp paired‐end runs.

### 2.4 Analysis and evaluation of variants

Sequence reads were mapped to the GRCh37/hg19 human reference genome. Variants were analyzed using four population databases (db‐SNP, genomAD browser, Exome Aggregation Consortium (ExAC), and 1000 Genomes Project Database) and three disease databases (OMIM, ClinVar, and HGMD). Variants were filtered if they had a minor allele frequency >0.5%, or >1% if they had been previously associated with disease (OMIM, ClinVar, and HGMD), or >2% if the variant was homozygous or if there was a second variant in the gene. Nonsynonymous variants and small indels in exons and splicing variants were selected to identify causal variants. Five applications (SIFT, Polyphen‐2, Mutation Taster, Mutation Assessor and CADD) were used to predict the potential impact of the identified mutations on protein function. We classified the variants in the cardiomyopathy‐associated gene according to the American College of Medical Genetics and Genomics (ACMG) guidelines (Richards et al., 2015).

### 2.5 Sanger sequencing

DNA samples from the proband and his parents were amplified, and the variants detected by whole‐exome sequencing were verified using Sanger sequencing with a 3730xl DNA analyzer (Applied Biosystems, Foster City, CA).

## 3 RESULTS

### 3.1 Clinical findings

**Figure 1:**
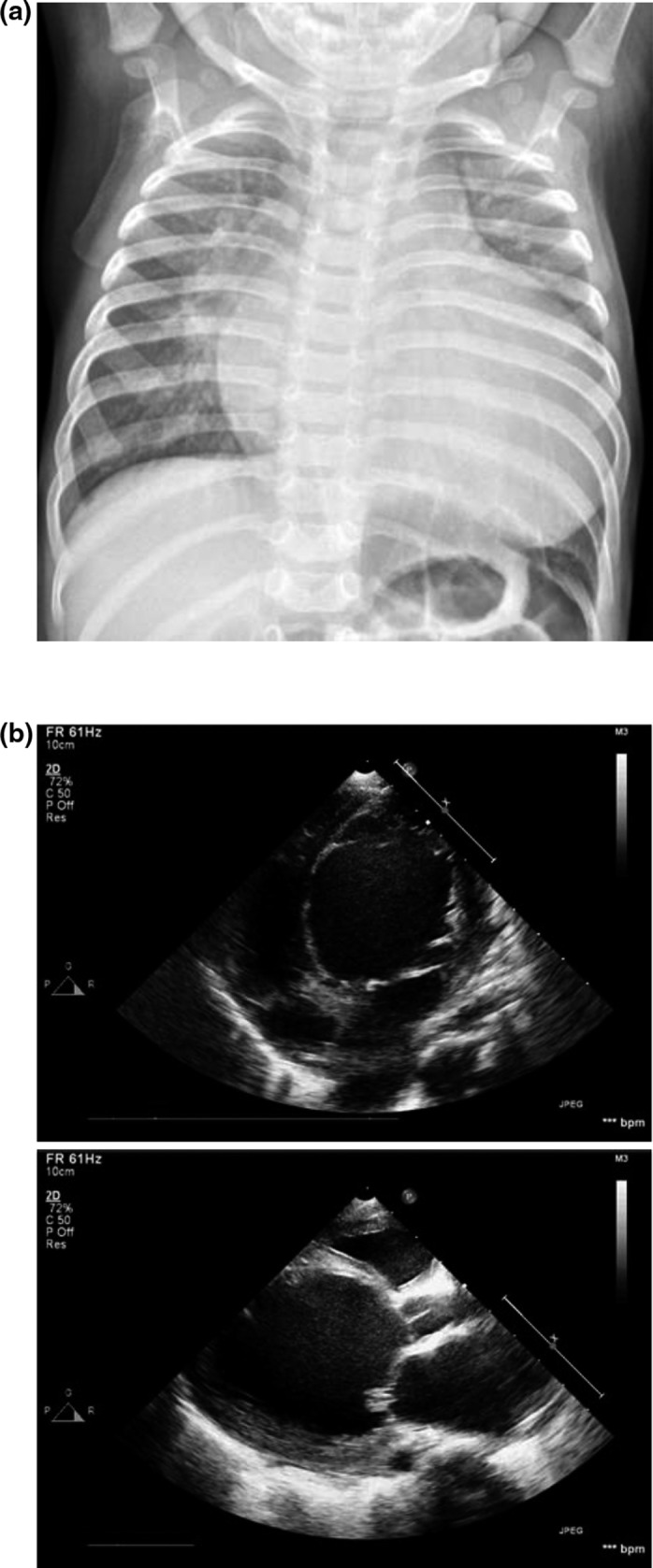
Radiographs of chest and echocardiographic examination of the proband. (a), the radiographs of chest displayed pneumonia and marked cardiomegaly with a cardiothoracic ratio of 0.69. (b), echocardiographic examination revealed a dilated left ventricle with prominent trabeculations, a depressed LV ejection fraction of 25%, and mild mitral regurgitation. The left atrial diameter was 20.3 mm. Right ventricular size, function, and wall structure were normal

The patient was an 11‐month‐old boy. He was born at 39 of weeks gestation by vaginal delivery with birth weight 3.5 kg and 49 cm length. The boy was admitted to a local hospital because of fever and cough. After 5 days of oral treatment with cefixime, his fever continued with persistent cough and diarrhea. Then he was transferred to our hospital. Radiographs of chest displayed pneumonia and marked cardiomegaly with a cardiothoracic ratio of 0.69 (Figure 1a). Echocardiographic examination revealed a dilated left ventricle (left ventricular diastolic dimension of 51 mm) with prominent trabeculations, a depressed LV ejection fraction (LVEF) of 25%, and mild mitral regurgitation. The left atrial diameter was 20.3 mm. Right ventricular size, function, and wall structure were normal (Figure 1b). ECG displayed sinus rhythm with ST‐segment changes. Serum biochemistry tests showed high levels of N‐terminal pro‐brain natriuretic peptide (NT‐proBNP; 17,108 pg/ml; normal range <300 pg/ml) and D‐dimer (1.77 mg/L; normal range <0.5 mg/L). The patient had no extracardiac manifestations and was diagnosed with pneumonia, DCM and LVNC. He died on the second day of hospitalization due to ventricular fibrillation.

The patient's parents are nonconsanguineous and there was no family history of cardiomyopathy and SCA. The mother had no complaints of abnormal manifestations during the perinatal period or history of reproductive system diseases.

### 3.2 Mutational analysis and Sanger sequencing

**Figure 2:**
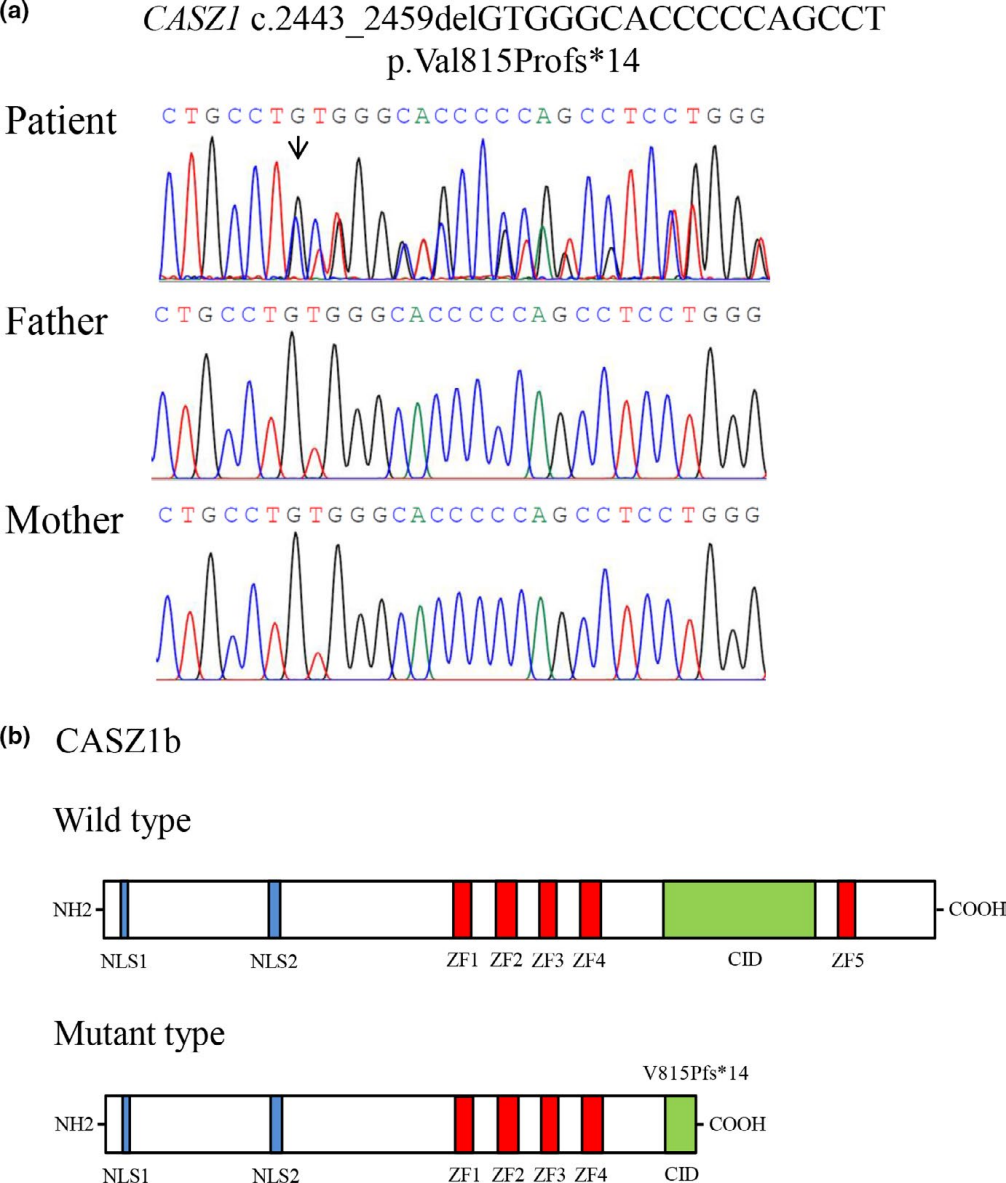
Sanger sequencing analysis and Schematic representations of protein structures. (a), Sanger sequencing analysis of *CASZ1* in the patient and his parents. The arrows indicated the mutated nucleotides. The patient carries a de novo heterozygous frameshift mutation (c.2443_2459delGTGGGCACCCCCAGCCT) in *CASZ1*. (b), schematic of the full‐length CASZ1b protein and V815Pfs*14‐mutant CASZ1 protein structures. NH2 means amino terminus; CID, CHD5‐interacting domain; COOH, carboxyl terminus; NLS, nuclear location signal; ZF, zinc finger

The mean coverage rate of target regions was 99.6% and on average 99.0% of the target bases were covered by >20× reads for each exome. The average sequencing depth of the exomes and their neighborhood was above 100×. A novel, de novo frameshift variant, c.2443_2459delGTGGGCACCCCCAGCCT (p.Val815Profs*14) of *CASZ1* was identified in the patient and was confirmed using Sanger sequencing in the proband and his parents (Figure 2a).

### 3.3 Pathogenicity evaluation of the identified variant

Human CASZ1 is expressed in two major alternatively spliced isoforms, CASZ1a and CASZ1b. CASZ1b is the more evolutionarily conserved isoform and most functional characterizations of mutant CASZ1 proteins were focused on the evolutionarily conserved CASZ1b isoform (Qiu et al., 2017). The frameshift mutation (c.2443_2459delGTGGGCACCCCCAGCCT, p.Val815Profs*14) of *CASZ1* in exon 11 is predicted to result in premature truncation of the protein, substituting valine at amino acid position 815 for proline. The severe truncation leads to a loss of 339 amino acids of CASZ1b, including zinc finger 5 and congenital heart disease 5 protein (CHD5)‐interacting domain (CID) of the C terminus of *CASZ1* (Sojka et al., 2014; Figure 2b). The probability of loss‐of‐function intolerance (pLI; Lek et al., 2016) for *CASZ1* in the ExAC Browser was 1.0, strongly supporting the fact that this variant is pathogenic. Neither the population databases nor the disease databases contain this variant. In line with the ACMG guidelines, the frameshift mutation c.2443_2459delGTGGGCACCCCCAGCCT of *CASZ1* can be classified as pathogenic.

## 4 DISCUSSION

The patient reported here is a carrier of a de novo frameshift mutation in *CASZ1*. He presented at the age of 11 months with DCM and LVNC. *CASZ1* contributes to VSD (Huang et al., 2016) and DCM (Qiu et al., 2017) in humans. However, none of the previously described patients carrying mutations in *CASZ1* has been reported to display LVNC as well.

CASZ1 is a para‐zinc‐finger transcription factor and localizes to the nucleus. CASZ1 is expressed in cardiomyocytes during the earliest stages of mammalian heart development and is essential for the proliferation of cardiomyocytes in the first and second heart fields (Dorr et al., 2015). CASZ1 has been shown to be important in cardiac morphogenisis and development in *Xenopus* (Christine & Conlon, 2008) and mouse (Liu et al., 2014). In *Xenopus*, failure of a subset of progenitor cells to differentiate into cardiomyocytes and aberrant cardiac morphogenesis was identified in CASZ1 depleted embryos (Christine & Conlon, 2008). Consistent with this, cardiac noncompaction and VSD were identified in *CASZ1* knockout mice (Liu et al., 2014). Abnormal expression of some *CASZ1* targeted genes, such as muscle contraction genes (*TNNI2*, *TNNT1*, and *CKM*), contractile fiber gene (*ACTA1*), and cardiac arrhythmia associated ion channel coding genes (*ABCC9* and *CACNA1D*), were identified in CASZ1 depleted embryonic hearts in mice, which might contribute to the heart defect (Liu et al., 2014). Furthermore, CASZ1 played an essential role in mammalian cardiomyocyte cell cycle progression (Dorr et al., 2015). Loss of CASZ1 leads to a marked reduction in DNA synthesis, an increase in phospho‐RB, and a decrease in the cardiac mitotic index due to a prolonged or arrested S phase (Dorr et al., 2015).

Recently, a *CASZ1* missense mutation p.L38P was identified in a congenital VSD family (Huang et al., 2016). However, a *CASZ1* nonsense mutation, p.K351X, was identified in a DCM family (Qiu et al., 2017). Functional studies revealed that both the L38P‐mutant and K351X‐mutant CASZ1 protein lost transcriptional activity (Huang et al., 2016; Qiu et al., 2017).

CASZ1 interacts with CHD5 and TBX20. CID of the C terminus of CASZ1 (amino acids 785–998) is necessary and sufficient to interact with CHD5. CHD5‐CASZ1 interaction is responsible for cardiac morphogenesis (Sojka et al., 2014). CASZ1 physically interacts with TBX20, and the four most N‐terminal zinc finger domains of CASZ1 are necessary for the CASZ1‐TBX20 interaction. Reduction or loss of physical interaction between TBX20 and CASZ1 leads to DCM (Kennedy et al., 2017). The frameshift mutation (c.2443_2459delGTGGGCACCCCCAGCCT, p.Val815Profs*14) was predicted to produce a truncated protein lacking the fifth zinc finger domain and the C terminus of CASZ1, including the CHD5‐interacting domain, but not the TBX20‐interacting domain. Nevertheless, the premature truncation of *CASZ1* likely mediates mRNA decay to disrupt the whole function of CASZ1. Furthermore, the ExAC pLI of 1.0 for *CASZ1* demonstrated that this gene is extremely intolerant of loss‐of‐function variation.

In the DCM family with *CASZ1* nonsense mutation p.K351X, the age at initial diagnosis of DCM was 25–45 years of age. However, our patient was diagnosed with DCM and HF at the age of 11 months, which was earlier than the patients reported. Furthermore, our patient displayed prominent trabeculations in the LV and was diagnosed with LVNC. The etiology of LVNC is uncertain, but it is speculated that it results from a disturbed compaction process during early development of the LV myocardium (Sedmera, Pexieder, Vuillemin, Thompson, & Anderson, 2000). *CASZ1* depletion in the murine has been shown to be implicated in the development of LV compaction during embryogenesis (Liu et al., 2014). Our study reveals *CASZ1* as a new causative gene responsible for human LVNC. It has been reported that subjects with LVNC and a dilated phenotype had higher rates of death or transplantation than matched subjects with DCM (Shi et al., 2018). The combination phenotype of DCM and LVNC may be explained the bad prognosis, including ventricular fibrillation and SCA of this patient.

In conclusion, we have identified a novel *CASZ1* variant in a patient with combined DCM and LVNC for the first time, thus broadening the phenotypic spectrum of *CASZ1* variants. Furthermore, this study emphasized the usefulness of whole‐exome sequencing for genetic diagnosis of cardiomyopathy.

## CONFLICT OF INTEREST

None declared.
